# Re-Evaluating the Transplant Glomerulopathy Lesion—Beyond Donor-Specific Antibodies

**DOI:** 10.3389/ti.2024.13365

**Published:** 2024-11-21

**Authors:** Arun Chutani, Daniel Guevara-Pineda, Gabriel B. Lerner, Madhav C. Menon

**Affiliations:** ^1^ Transplant Nephrology, Yale University of School of Medicine, New Haven, CT, United States; ^2^ Nephrology, Yale University, New Haven, CT, United States; ^3^ Renal Pathology, Yale University, New Haven, CT, United States; ^4^ Nephrology, Medicine, Research in Kidney Transplantation, Faculty in Human Translational Immunology and Translational Biomedicine, Yale School of Medicine, New Haven, CT, United States

**Keywords:** transplant glomerulopathy (TG), anti HLA antibodies, banff criteria, podocyte, C4d

## Abstract

There have been significant advances in short-term outcomes in renal transplantation. However, longer-term graft survival has improved only minimally. After the first post-transplant year, it has been estimated that chronic allograft damage is responsible for 5% of graft loss per year. Transplant glomerulopathy (TG), a unique morphologic lesion, is reported to accompany progressive chronic allograft dysfunction in many cases. While not constituting a specific etiologic diagnosis, TG is primarily considered as a histologic manifestation of ongoing allo-immune damage from donor-specific anti-HLA alloantibodies (DSA). In this review article, we re-evaluate the existing literature on TG, with particular emphasis on the role of non-HLA-antibodies and complement-mediated injury, cell-mediated immune mechanisms, and early podocyte stress in the pathogenesis of Transplant Glomerulopathy.

## Introduction

Transplant Glomerulopathy (herein referred to as “TG”), by definition, is a morphologic description of histologic or ultrastructural alterations and not a specific clinicopathologic entity. The hallmark morphologic manifestation of TG is glomerular basement membrane double contouring, either by light or electron microscopy [1]. The term was initially described in detail by Zollinger *et al* in 1973 [2]. TG is a contributing factor in early (less common) and, particularly, late allograft loss. TG lesions, when encountered in allograft biopsies, have been repeatedly associated with poor graft outcome [3], but studies have so far not revealed any specific therapeutics that reverse or even improve outcomes in TG [3–5].

## Incidence and Epidemiology

Studies from the French-Canadian cohort have informed the community regarding the cumulative incidence of post-transplant TG. In this study, TG was present in 5% of the 1-year protocol biopsies and was associated with poorest survival [6]. A second study reported the progressively increasing prevalence of TG with transplant vintage: 2.8% at 1 year, 6.1% at 2 years, 8.5% at 3 years, and 11.5% at 5 years [7]. This larger study from the Mayo clinic involving 582 surveillance biopsies collected over 5 years found a mean prevalence of TG near 10% with a cumulative incidence of 20% by 5 years. In this study, TG was associated with both acute and chronic Banff lesions, albeit milder TG (Cg = 1) was more prevalent in early surveillance biopsies (1 year) and cross-sectionally less associated with Banff chronic lesions, while higher Cg scores were more common in later surveillance biopsies. In general, higher Cg scores in a biopsy was associated with greater numbers of glomeruli samples in the biopsy being involved with TG. In a retrospective review (unpublished) of our institutional database since 2000, 189 cases with TG diagnoses were detected, which provided an incidence of TG at near 3% of allograft biopsies per year.

When TG occurs in biopsies, it has independently been associated with allograft loss [7]. A study examining causes of graft loss censored for death identified TG as the potential driving lesion in ∼15% of such cases [8]. Recent meta-analyses, including >5,000 published patients, reaffirmed the increased risk of graft loss with a diagnosis of TG, potentially independent of time from transplantation, showing an estimated difference of median survival of nearly 12 years in patients with TG lesions vs. those without [9]. The prognosis between subclinical and clinical TG has also been reported to be similarly poor. However, as discussed below, within the TG diagnosis, multiple sub phenotypes and clusters may attenuate or aggravate the outcome associated with TG. Hence, together, these studies establish an early prevalence of the TG diagnosis within year-1 of near 5%, followed by a slower increase in the incidence per year of TG related to time from transplantation [7] and an association with increased risk of graft loss with the development of TG.

## Histology of TG: Clinico-Pathologic Correlations and Prognosis

**FIGURE 1:**
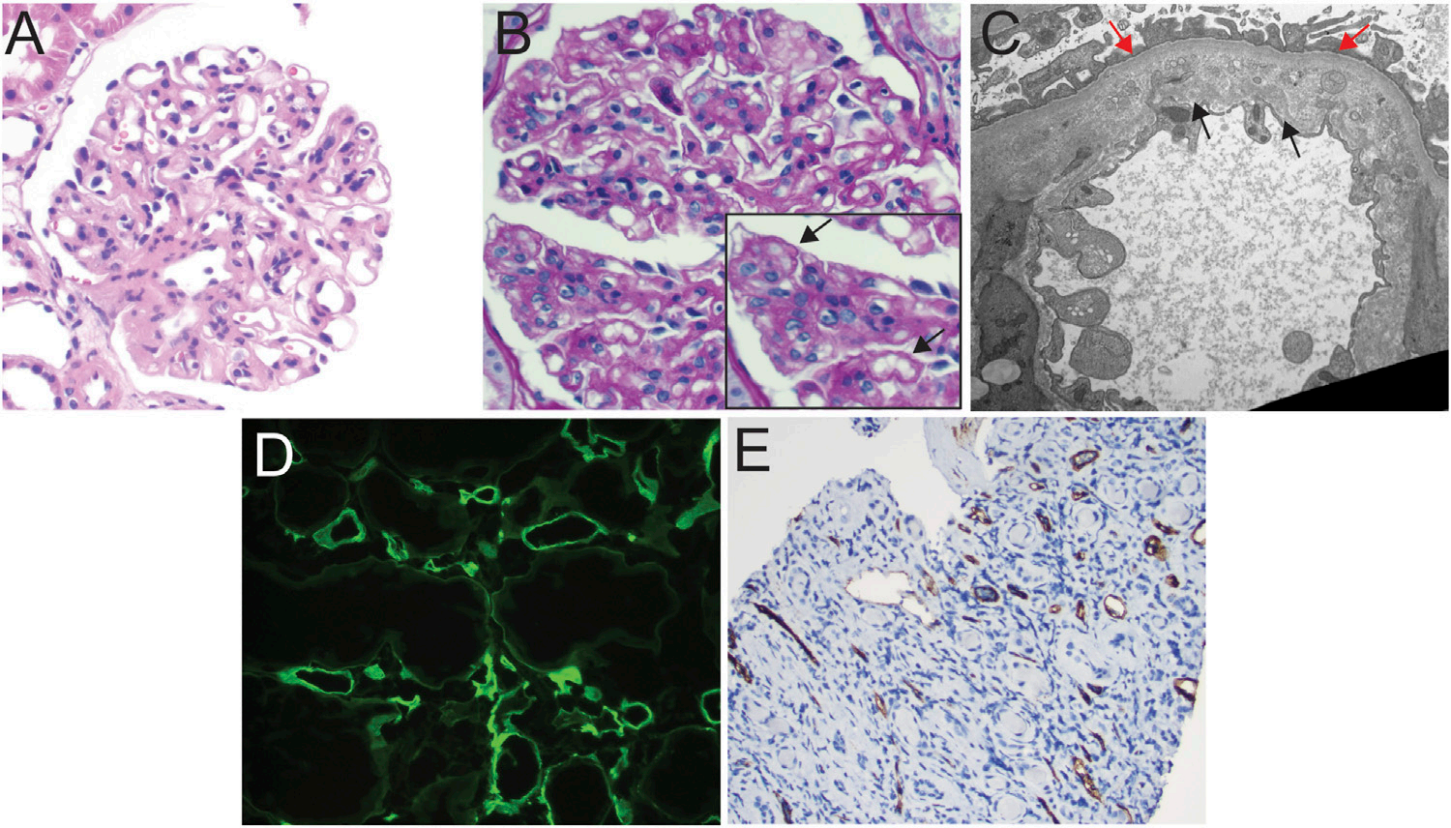
Light microscopic and ultrastructural findings in allograft biopsies demonstrating C4d staining and transplant glomerulopathy. **(A)** Hematoxylin and eosin stain of allograft biopsy with glomerulus showing global wall thickening and increase in mesangial cellularity, suspicious for transplant glomerulopathy, 200x. **(B)** PAS stain of glomerulus demonstrating reduplication of the capillary basement membranes (see inset, black arrows), 200x. **(C)** Transmission electron photomicrograph showing expansion of the lamina rara interna by grungy variably electron-dense material, as well as areas of original (red arrows) and *de novo* (black arrows) basement membrane formation, indicative of transplant glomerulopathy, TEM, 13,900x. **(D)** Immunofluorescent staining for C4d showing diffuse circumferential positive staining in peritubular capillaries in a patient with concurrent transplant glomerulopathy on biopsy (patient developed positive Class II donor-specific antibodies ∼2 years after diagnosis; 100x). **(E)** immunohistochemical staining for C4d showing diffuse positive staining in peritubular capillaries in a patient demonstrating early transplant glomerulopathy and findings indicative of chronic antibody-mediated rejection on biopsy, 100x.

Clinically, patients with biopsy-proven TG tend to show proteinuria, edema, a slowly rising creatinine, and shortened allograft survival. However, TG is a pathologic entity defined primarily as reduplication/multilayering of the glomerular basement membrane (Figures 1A, B) as observed by light and/or electron microscopy in the kidney allograft, in the absence of immune deposits. According to current Banff schema, either TG or peritubular capillary multilayering (*ptcml*) are required features in the diagnosis of chronic active antibody-mediated rejection (cABMR) [10]. TG is defined using the *cg* scoring system, with a lesion Score *cg >* 0 requiring *de novo* basement membrane formation in at least three capillary loops by EM (cg1a), or double contours in only a single capillary loop as the minimal light microscopic finding (cg1b) after the exclusion of chronic thrombotic microangiopathy, recurrent, or *de novo* Glomerulonephritis (Table 1; Figures 1B, C). The *ptcml* lesion, defined ultrastructurally by seven or more peritubular capillary basement membrane layers in a single capillary, or five or more in at least two peritubular capillaries, has been shown to correlate with worse overall prognosis in cases of TG [11]. An increase in the mesangial matrix may be present but is less specific and is not required for diagnosis. TG can be differentiated from the recurrent and *de novo* glomerulonephritis by negative Immunofluorescence for IgG, IgA, IgM, C1q, C3, *κ*, and *λ* and absence of immune complex deposition [12]. Mesangiolysis and glomerulosclerosis may be present, mimicking focal segmental glomerulosclerosis, while microvascular inflammation, including glomerulitis (g) and/or peritubular capillaritis (ptc), may be observed in cases mediated by alloimmune mechanisms [13, 14]. Glomerular C4d is found in lesions associated with TG, and the strength of its association increases when associated with peritubular capillary C4d (Figures 1D, E). [15]. Glomerular C4d positivity is not a part of the current Banff criteria, i.e., C4d should be evaluated in peritubular capillaries or vasa recta only. Glomerular C4d staining could be a tool to detect a humoral etiology in cases of DSA-negative microvascular inflammation, if further validated [15–18]. Using electron microscopy (EM), features of TG can be identified as those of endothelial injury, whose definition was refined as endothelial cell enlargement, subendothelial electron-lucent widening, and subendothelial neo-densa glomerular basement membrane formation [16].

**TABLE 1:** Banff quantitative criteria for transplant glomerulopathy (cg) (Adapted from Roufosse et al [1]).

cg0	No glomerulopathy. Double contours in <10% of peripheral capillary loops in most severely affected glomerulus
cg1	Double contours affecting up to 25% of peripheral capillary loops in the most severely affected non-sclerotic glomerulus. If noted by EM only, *cg1a* is assigned; if by light microscopy, *cg1b* is assigned
cg2	Double contours affecting 26%–50% of peripheral capillary loops in the most affected non-sclerotic glomerulus
cg3	Double contours affecting >50% of peripheral capillary loops in the most affected non-sclerotic glomerulus

It is imperative to consider that TG changes are primarily observed on the lamina rara interna side of the GBM, indicating the role of endothelial side injury. These findings are similar to other entities associated with endothelial injury, such as chronic thrombotic microangiopathy (cTMA). Electron-dense deposits or characteristic immunofluorescence findings are not present in TG and, if present, would suggest a recurrent or *de novo* immune complex glomerulonephritis, especially immunoglobulin-mediated (type 1) membranoproliferative glomerulonephritis or a C3 glomerulopathy. TG is diagnosable in the most recent Banff criteria purely by EM in the absence of double contours by light microscopy. This requires glomerular basement membrane duplication in at least three glomerular capillaries and is designated cg1a by Banff criteria (see Table 1).

While the diagnosis of TG relies on a semiquantitative score identified by either light microscopy and/or EM, it oversimplifies a rather complex phenotype resulting from multiple etio-pathogenetic mechanisms. For instance, a retrospective study of 209 allograft biopsies performed for chronic allograft dysfunction identified 25 which met the pathological criteria of TG. Three partially overlapping etiologies accounted for 21 (84%) cases: C4d-positive (48%), hepatitis C-positive (36%), and chronic thrombotic microangiopathy (cTMA)-positive (32%) TG. The majority of patients with confirmed cTMA were also hepatitis C-positive, and the majority of hepatitis C-positive patients had TMA [19]. However, with the success in curing hepatitis C with anti-viral agents, HCV-related TG is expected to be insignificant in the modern context.

Pathologic features encountered in TG have also been associated with prognosis. Using a cohort of 92 patients with TG, of whom 64 developed allograft failure within 5 years of diagnosis, a prognostic index (PI) utilizing component Banff lesion scores was developed and then validated in an independent external of kidney transplant recipients with TG [20]. In this elegant study using principal component analyses, a “chronic-inflammation” score combining Banff ci, ct, and ti scores during TG was associated with time-dependent risk to graft failure. Interstitial fibrosis and tubular atrophy (Ci + Ct) have been consistently associated with poor kidney outcomes. In a recent analysis aimed at evaluating the histological risk factors for kidney allograft loss, the key finding was that, regardless of the specific cause for chronic histological damage, the presence of interstitial fibrosis and tubular atrophy had an additive and independent impact on graft outcomes, including those patients with TG [21]. One study evaluated 147 cases of either active or cABMR using activity and chronicity indices and found that a chronicity index score of 4 or greater (including components of cg score, as well as ci, ct, and cv scores) was associated with worse overall graft survival, independent of other parameters [22]. Others, however, have called into question the reproducibility of such a chronicity index [23]. Indeed, reproducibility and interobserver variability in feature identification, including the cg score, are known issues in the field of allograft pathology [24–27]. Overt evidence of DSA-mediated mechanisms on serology/pathology have shown distinct prognostic associations. In a case series of 71 patients with TG, Lesage *et* al [28] noted that eight were donor-specific antibody positive/C4d negative, six were donor-specific antibody negative/C4d positive, and only 12 were donor-specific antibody positive/C4d positive. The long-term outcomes for all three groups were similar and significantly worse than those with both Cd and donor-specific antibody negativity [28]. Serum creatinine and proteinuria at the time of biopsy have also shown to be independent risk factors for graft survival in TG, signifying co-occurrence of cumulative damage [29, 30].

## Potential Pathogenetic Mechanisms Related to TG

**FIGURE 2:**
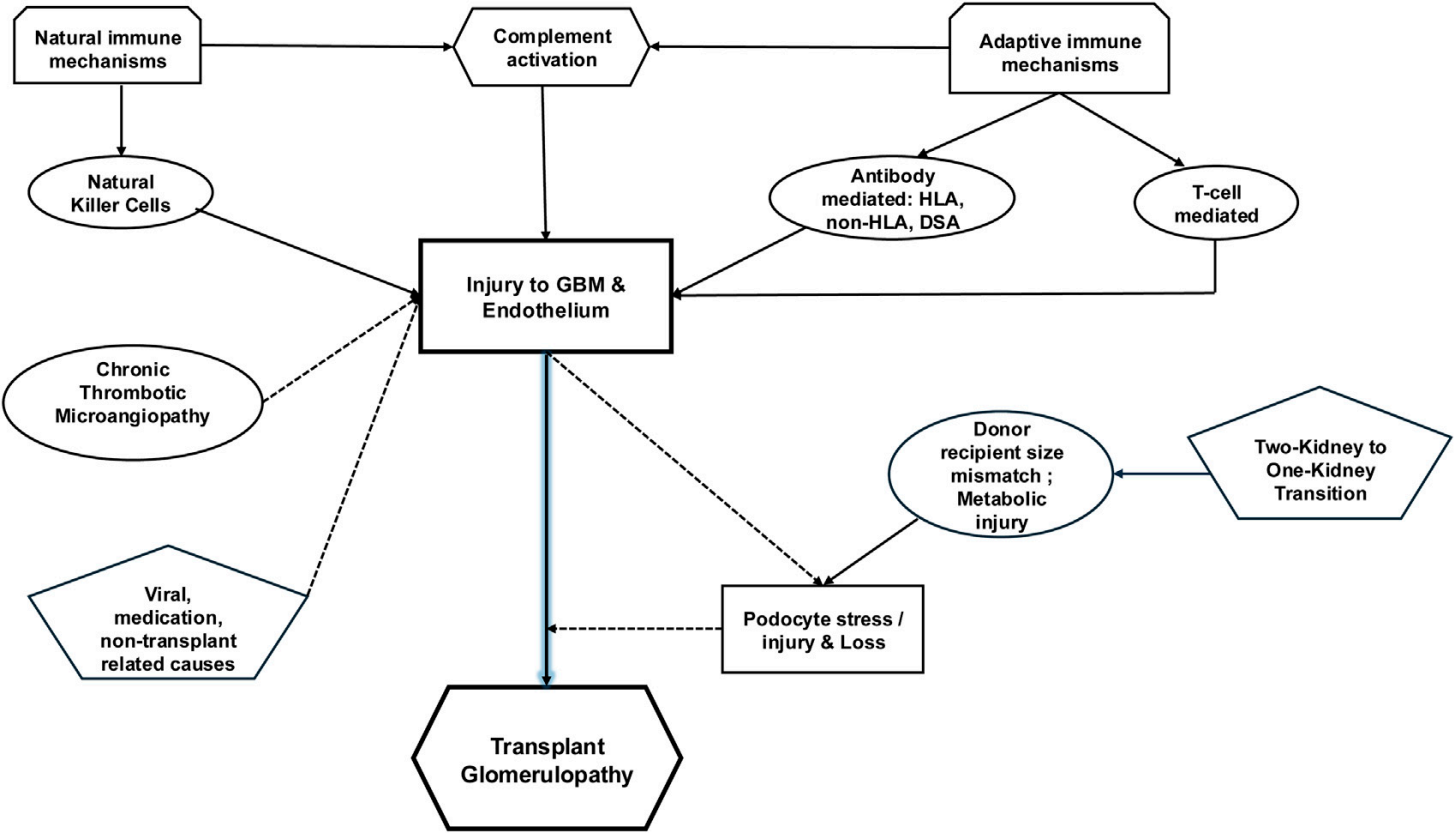
Schematic of potential pathogenetic mechanisms related to TG. TG defined by basement membrane multilayering on the lamina rara densa side is likely initiated by immunologically mediated endothelial injury. While the role of DSA is frequently reported, selected anti-donor antibodies directed against non-HLA proteins have been incriminated. Accompanying complement mediated injury likely plays a role, along with anti-donor antibodies. A proportion of TG is unassociated with DSA and is now attributable to innate and adaptive cell-mediated mechanisms. Activated NK cells seem to be associated with TG in combination with DSA, while T-cell infiltration/activation has been observed without DSA. An understudied contributor to TG is the role played by podocyte stress from obligate glomerular hypertrophy, compounded by donor-recipient weight mismatch and metabolic or immunologic injury. Whether podocyte loss is causal in TG remains a hypothesis in need of testing. Endothelial injury from other sources (cTMA, viral injury, or medications) can cause/compound endothelial injury and multilayering.

Our understanding of TG continues to evolve. Many active players identified are associated with TG and are potentially incorporated as pathogenetic factors, including HLA and non-HLA antibodies, donor-specific antibodies (DSA), cell-mediated mechanisms, and podocyte stress [summarized in Figure 2 schema]. In this section, we will discuss in greater detail the role and extent of the scope of each of these entities.

### Anti-HLA-DSA, Chronic Antibody-Mediated Rejection (cABMR), and TG

Numerous studies identify the role of DSA in TG [31–35], and TG is included in the constellation of findings that constitutes cABMR in Banff schema [16]. In our data, we observed several cases from our institution with concurrent TG and evidence of cABMR (Figure 1). While class I [34, 36–41] and Class II DSA-positive cases have been associated with TG [7, 34, 36, 42–44], a role for HLA Class II DSA (whether pre-transplant and *de novo*) with TG pre-eminently emerges from literature [36, 45–47]. For instance, in a study evaluating sub-phenotypes of TG from 1,036 indication biopsies, where TG was diagnosed in 53 (5.1%) cases at a median of 5 years post-transplant, the frequency of circulating anti-HLA alloantibody (both DSA and non-DSA) were connected to peritubular capillary basement membrane multilayering, peritubular capillary deposition of C4d, and double contouring of glomerular basement membranes [48]. Peritubular capillary basement membrane multilayering was present in 48 (91%) and C4d staining was detected in 18 (36%) cases of TG. The presence of anti-HLA antibodies was detected in 33 (70%), among whom 28 (85%) were DSA. Among anti-HLA antibodies, those directed against Class II (13/33) or against class I and II (17/33) were more common than those against class I (3/33) antigens. Thus, from this dataset of biopsies obtained for cause, 70% of TG has potential evidence of antibody-mediated phenomena—especially Class-II DSA. Furthermore, studies also suggest reduced 5-year graft survival with class II sensitization over Class I [36], and the presence of TG at 1 year after transplantation resulted in graft loss of approximately 30% in the former vs. 20% in Class-I-positive patients [36]. Within Class II, anti-DQ DSA has been increasingly associated with the development of TG [46, 49–51]. Coemans et al in 2021 performed a single-center cohort study including 2,761 protocol and 833 indication biopsies. Patients with pretransplant HLA-DSA were more prone to develop histology of acute antibody-mediated rejection, TG histology, or a combination of both. This manuscript provided a detailed statistical analysis of the causal relationship between HLA-DSA and TG and showed that the deleterious graft outcomes of HLA-DSA are mediated by the occurrence of AMR and transplant glomerulopathy [52].

Together, these data point to the concept that, while DSA are associated with TG, nearly 30%–40% of TG lesions must develop from different pathogenetic mechanisms [48]. Analogously, not all patients with detectable class II DSA develop TG, suggesting the need for additive/alternate injury mechanisms. Indeed, traditionally, peritubular capillary basement membrane multilayering has been considered a lesion associated with repeated endothelial injury by DSA; however, in our recent work, we reported that these lesions were identifiable at a similar rate in patients with or without DSA but associated with microvascular inflammation (MVI), suggesting that the presence of endothelial injury may be causative rather than the specific type of injury associated with DSA [53].

### C4d Staining in Transplant Glomerulopathy

In the complement cascade, C4d is generated from C4 following activation of C1Q via DSA or polysaccharides in the mannose binding lectin pathway [4, 54]. Complement activation in the presence of DSA (HLA or non-HLA) may play a role in TG. However, DSA- and C4d-negative TG is well recognized [36, 41]. Similarly, studies of TG report C4d-negative but DSA-positive cases [4, 36], suggesting these two features are not always correlated in TG. It must be noted that C4d is covalently bound to the tissues and, while considered a footprint of prior antibody activity, C4d positivity may remain after injury [4, 46, 55] or indeed be evanescent [4]. It is also important to note that the rate of C4d detection of TG in reported studies is also contingent upon the technique used in a particular dataset, whether immunofluorescence or immunohistochemistry [56]. Pathogenetically, the hallmark lesion of TG is likely endothelial cell injury with swelling, loss of fenestrations leading to sub endothelial widening of lamina rara interna and electron lucent or flocculent material, cell debris and reduplication, or multi layering of lamina densa [4]. Electron dense deposits, which are the *sine qua non* of recurrent or *de novo* immune complex glomerulonephritis in the allograft (immunoglobulin mediated or C3 glomerulopathy), are not encountered in TG and show that complement activation within the glomerulus is not sufficient to induce a TG phenotype in these other glomerulitides.

### Transplant Glomerulopathy in the Absence of DSA: Adaptive Immune-Cell-Mediated Mechanisms

Several studies have now demonstrated that DSA is neither necessary nor sufficient to induce the lesions of TG. Vongwiwatana *et al* [57], showed that only 25% of biopsies with TG had associated positive C4d deposition. Similarly, in a study by Aly *et al* [58], all 20 patients with TG were C4d negative. It must be noted here that DSA + C4d-negative TG biopsies were similar to cABMR in their gene-expression profile, suggesting that DSA-mediated injury can occur without detectable C4d [59]. In this latter study by Akalin *et al*, a substantial number of patients with TG did not have either positive C4d staining or DSA [60], while another retrospective study of TG showed that only 45% had detectable DSA and only 14% were C4d-positive. Indeed, demonstration of sub-phenotypes within TG using DSA also highlights non-DSA-mediated TG. Lower glomerulitis and peritubular capillaritis scores, less C4d deposition, and less interstitial inflammation have been seen in the absence of DSA compared with DSA–positive biopsies. Analogously, the severity of Banff *g, C4d,* and *i* scores were less pronounced in the absence of HLA-DSA [29], although the distribution of the chronic lesions and the graft function, assessed by serum creatinine, proteinuria, and eGFR, were comparable between the HLA DSA–negative and HLA-DSA–positive TG phenotypes [29]. Hence, when applying conventional markers of DSA-mediated allograft injury (i.e., serologic DSA or C4d), the pathogenesis of many cases of TG remains unexplained. Novel markers have suggested that cell-mediated mechanisms, rather than DSA, underlie TG in a significant proportion of cases. Dean et al. compared intragraft biopsy gene expression profiles between positive cases crossmatch-associated with TG, non-DSA-TG, and 10 conventional DSA-neg controls with stable histology [61]. Despite the antibody involvement, gene expression profiles associated with cell-mediated immunity were significantly enriched, including those for cytotoxic T lymphocytes and Interferon Gamma [61]. Similarly in six TG biopsies, glomerular staining for the costimulatory molecule ICOS and the chemokine receptor CXCR3 with its ligand Mig were present, indicating the presence of activated T cells in DSA-negative TG.

Innate immune-cell-mediated mechanisms: A role for NK cell-mediated recognition of “non-self” donor tissue and consequent injury, independent of development of DSA, has also been shown in MVI cases [62, 63] without demonstrable DSA. Notably, the association of NK cells with the development of TG in this form of injury is not as well established in current human data. Pioneering animal studies of ABMR using CCR5-knockout-B6 mice as kidney recipients show that depletion of NK cells prevented acute ABMR lesions despite the presence of high DSA titers. However, longer follow up of these allo-transplants allowed the development of TG, suggesting that NK cells may mediate the ultimate TG phenotype when combined with DSA. In this model, high levels of IFN-y, perforin, and granzyme B were found 3 days after transplantation, suggesting T cell/NK cell activation in the allograft occurred even before DSA was detectable in recipient mice with MVI [64]. A recent study compared bulk transcriptomics of 15 cABMR biopsies (all with evidence of TG), 17 T-cell mediated rejection (TCMR cases without Cg), and 18 non-rejectors (NR) [65]. The study found marked enrichment of NK-cell activation signatures in cABMR vs. NR biopsies, which were confirmed by deconvolution analyses. While both TCMR and cABMR showed NK cell enrichment, this was highest in the cABMR transcriptomes. Another recent study delineated differential roles of NK- and T-cell mediated injury in subtypes of TG cases [66]. The authors compared the transcriptomic profiles of 14 TG cases without DSA/C4d with 22 cases classified as TG with DSA^+^/C4d^+^ using the NanoString Banff-Human Organ Transplant (B-HOT) panel with subsequent multiplex immunofluorescence validation. DSA^+^/C4d^+^ TG showed a higher glomerular abundance of natural killer cells/macrophages and increased expression of complement-related genes and DSA-related pathways vs. samples DSA^−^/C4d^−^ TG, while the latter showed enrichment of genes related to the activity of T cells (CD3^+^, CD8^+^). At the protein level, using multiplex immunofluorescence, they confirmed increased T-cell markers (CD3^+^, CD3^+^CD8^+^) in DSA^−^/C4d^−^ TG. Together, these data suggest that T-cell- and NK cell-mediated rejection phenomena are independently or co-operatively involved in the pathogenesis of TG.

### Role of Non-HLA Antibodies

Non-HLA antibodies against polymorphic donor antigens can result from classical adaptive allo-immune responses initiated by T-cells culminating in specialized antibody-secreting plasma cells but can occur as part of the autoimmune phenomena or naturally occurring IgG. Ischemia-reperfusion injury and/or episodes of rejection may unmask polymorphic donor antigens causing antibody formation against non-HLA proteins [67]. Non-HLA antibodies clearly contribute to ABMR in HLA identical renal allografts exemplified by anti-donor endothelial reactive antibodies [68]. Recent evidence suggests non-HLA auto- and alloantibodies (through independent cytotoxicity or with concurrent DSA) contribute to TG (reviewed in [3, 69]). Interestingly, the most widely studied non-HLA antigen target implicated in ABMR is the angiotensin receptor (AT1R). However, the association of anti-AT1R antibodies with TG independent of HLA-DSA is still unclear [70, 71].

Dinavahi et al [72] used protein microarrays to compare antibody panels in pre- and post-transplant sera from patients with and without transplant glomerulopathy and saw reactivity against peroxismal-trans-2-enoyl-coA-reductase being associated with development of TG. In mouse models, the development of antibodies to glomerular basement membrane components, such as perlecan or collagens type IV or type VI, has been associated with the development of TG [73]. In clinical data, the presence of antibodies against the glomerular basement membrane components, particularly anti-Agrin antibodies, were identified in 44% of TG cases (16 patients) and associated with rejection episodes prior to the diagnosis of TG [74]. Another group identified that antibodies to renal tissue restricted self-antigens, particularly fibronectin and collagen type IV, increased the odds of TG [75]. Here, non-HLA antibodies were detected irrespective of the presence of anti-HLA antibodies, while MVI features and C4d deposition were equivalent in patients with non-HLA or HLA-antibodies, suggesting an independent role for non-HLA antibodies in the development of TG. In a highly sensitized cohort (75% DSA-positive), anti-endothelial cell antibodies (AECAs) detected by ELISA were demonstrably increased with ABMR along with MVI lesions and TG [76]. This suggests that non-HLA antibodies are associated with TG clinically and experimentally, but a focused approach examining antibodies specifically against polymorphic or self-restricted endothelial or GBM antigens, restricted to patients with suspected antibody-mediated (C4D-positive) TG, may be required to reveal potential culprits. A major limitation for non-HLA antibody detection is the limited utility of commercial panels currently available for this purpose.

### Role of Podocyte Stress in TG: Cause, Effect, or Guilt by Association?

The stereotypic lesions of TG do not develop in usual contexts of glomerular injury in binephric individuals, suggesting a unique predilection for the uninephric allograft state. Although endothelial damage likely initiates the cascade of immunologic injury culminating in characteristic TG, a role for increased podocyte stress leading to critical podocyte depletion [77] and TG is evolving. Notably, advanced TG is characterized by significant proteinuria, progressive injury, and graft loss similar to podocytopathies. To study this aspect of the development of TG, Wiggins et al, tested the role of the “two kidneys to one kidney transition” that occurs in all allografts and resultant podocyte stress using urine podocin/creatinine ratio as a marker of podocyte injury/depletion [78]. Surprisingly, while allograft recipients have half the number of nephrons, they observed a significantly increased rate of podocin mRNA excretion (a surrogate of podocyte loss) in urine of all allograft recipients vs. binephric controls and that urinary podocyte loss was markedly higher in patients with TG vs. patients with non-glomerular allograft pathology (tubular injury or IFTA). The steepest decline in podocyte density occurred in the subset of TG patients diagnosed within 2-year of transplant (early TG). Later stage TG (>2 years) was less associated with biopsy-confirmed rejection episodes and had a distinct podometric profile [78]. The same group reported that such early loss of podocytes in urine from any cause was associated with later graft loss [79]. This was exacerbated in context with donor-recipient weight mismatch, which is a surrogate marker for glomerular stress and suggests a greater need for post-transplant compensatory hypertrophy [78, 80, 81]. Another potential extrapolation of this inference is that post-transplant obesity and metabolic syndrome in the context of immunosuppression could be a predisposing factor in exacerbating podocyte loss, leading to accelerated loss of graft function and early onset of TG. Additionally, the same researchers also reported that, even within normal ranges of mean arterial pressures (MAP), increases in MAP were linearly associated with urine markers of podocyte stress [82], bringing out a role for fluid-flow and shear stress [83]. In this pathogenetic chain, the specific role played by immunologically mediated endothelial injury, leading to exacerbation of podocyte stress, still needs to be better defined.

While associative data shows podocyte damage or podocyturia in the context of TG, it must be noted that there is no conclusive evidence directly linking podocyte damage as a causal factor in TG. Most of the current evidence supports the hypothesis that TG is initially characterized by glomerular basement membrane alterations and endothelial injury, which could then lead to podocyte injury with foot process effacement or podocyte loss. The specific causal role of podocyte loss in TG, if any, remains an area in need of mechanistic research.

### Non-Transplant-Related Contributors in GBM Multilayering

Role of hepatitis C: Hepatitis C is associated with immune-complex-mediated MPGN, where histological findings may overlap with TG (reviewed in [4]). It is not entirely clear whether alterations in morphological appearance of usual MPGN result from concurrent immunosuppression with altered immune complex deposition or a uninephric state following transplantation. It is postulated that hepatitis C could upregulate alloimmune responses and microvascular inflammation, leading to C4d positivity [4, 84]. HCV-associated injury, especially cryoglobulinemia, could also induce endothelial injury and histologic features of thrombotic microangiopathy (TMA), all contributing to TG-like lesions. With effective HCV treatment, such co-occurrences will be increasingly infrequent.

Chronic Thrombotic microangiopathy: cTMA with resultant endothelial injury may produce electron and light microscopic lesions, which may be very difficult to distinguish from TG [4, 84]. Clinically, cTMA has characteristic serologic findings, including microangiopathic hemolytic anemia and thrombocytopenia on peripheral blood smear, suggesting intravascular hemolysis and thereby differentiating it from TG.

## Conclusion

In summary, in this review article we discuss potential patho-mechanistic processes that have been elucidated by recent research to be in the evolution pathway of TG. While primarily understood to be initiated after persistent injury to the allograft glomerular endothelium from serologic- (DSA, non-HLA antibodies, and/or complement-mediated) or cell-mediated mechanisms, subsequent co-incidental podocyte stress and detachment (hypertrophic, hypertensive, and/or immunologic) could contribute as a common pathway leading to critical podocyte loss, often associated with increasing proteinuria over the course of graft life-span and in turn leading to progressive graft damage. Since podocytes have recently been ascribed a buttressing function in homeostatic and injured glomeruli [85–87], their contributory vs. potential causative role in TG needs to be better defined. A current problem in the field is the limited availability of long-term allo-transplantation models in transgenic rodents that also develop TG lesions. Rodent models of TG could help investigate cell-type- and gene-specific- modulations to differentiate causation vs. association as it relates to podocytes. On the other hand, non-human primate allo-models which do develop TG-like lesions in the context of DSA-mediated injury have not yet specifically focused on podocyte injury—again a deficiency in the field. Hence, mechanistically focused research is needed to unravel molecular targets that could be utilized to arrest the progression of TG. Ultimately, a multi-pronged approach targeting both initiators and effectors of TG will be needed to combat this important contributor to delayed graft loss.
